# Institutional incentives for altruism: gifting blood in China

**DOI:** 10.1186/1471-2458-13-524

**Published:** 2013-05-30

**Authors:** Chengpu Yu, Eleanor Holroyd, Yu Cheng, Joseph Tak Fai Lau

**Affiliations:** 1 Center of Medical Anthropology and Behaviour Health, School of Sociology and Anthropology, Sun Yat-sen University, Guangzhou, China; 2 Division of Nursing and Midwifery, RMIT University, Melbourne, Australia; 3 School of Public Health and Primary Care, The Chinese University of Hong Kong, Sha Tin, Hong Kong

**Keywords:** Blood collection, Blood donation, Institutional incentives, China

## Abstract

**Background:**

In mainland China, the motivation behind voluntary blood donation is a relatively new and understudied behavior. In recent times provincial governments in China have implemented various institutional incentive measures. However, little is known regarding the effectiveness of such measures. This qualitative study investigated the nature and outcomes of some identified institutionalized mechanisms, in particular how these were created and distributed in the form of incentives for voluntary blood donation.

**Methods:**

Participatory observations were conducted at two blood donation stations and four blood collecting vehicles in Changsha city, China. In-depth interviews were conducted with 17 staff and 58 blood donors at the aforementioned venues from May to October 2008 in Changsha.

**Results:**

Thematic analysis revealed the operation of four primary type incentives: policy-driven, symbolic, information feedback and role models, which constituted the system of institutional incentives. The current blood reimbursement system was not the primary motivation for blood donation; instead this system was a subtheme of future assurance for emergency blood needs. It was evident that symbolic incentives stressed the meaning and value of blood donation. Furthermore, post-donation information services and the inherent mechanisms of communication, enhanced by some public role models, served to draw the public to donate blood.

**Conclusions:**

At the institutional level, blood donation was not only informed by altruism, but also carried a system of benefit and reward for the donors and their family members. We would recommend that such arrangements, if accommodated effectively into China’s health promotion strategies, would increase the likelihood of blood donation.

## Background

In mainland China, voluntary blood donation is a relatively new and understudied behavior. Blood has deep cultural meanings in China as it is seen to be very precious. Losing blood is equivalent to losing one’s bodily vigor or ‘Yuan QI’ [1]. Blood is also seen as a gift from one’s parents and it is against filial piety [1,2] to give it away. These traditional Chinese beliefs may discourage voluntary blood donation. Hence, it is important to instill various types of incentives to counteract such cultural disincentives.

Historically, contaminated commercial blood/plasma donations have caused HIV outbreaks among many villagers in some provinces in China [3-5]. The crisis prompted the Chinese government to amend its blood donation laws and policies in 1997 and to promote voluntary blood donation actively. Currently, voluntary blood donation has become the sole source of blood supply for clinical use in most cities in China. New measures entitle blood donors and their family members to a lower-cost or free life-time blood transfusion when needed, proportionate to the volume of blood donated.

A recent literature review showed that recent studies tend to use psychological theories or related factors to explain and to promote blood donation [6]. Positive factors motivating voluntary blood donation include altruism, incentives, as well as influences exerted by one’s significant others and the press. Negative factors include fear, short-term donor deferral (e.g. low hematocrit, sore throat and fever), permanent deferral and medical reaction (e.g. physical reactions to blood donation) and widespread disinterest [7]. It is contended that external motivation drives people to donate blood for the first time; internalized motivation (e.g. the virtue of donation and the norm of helping others) is then formed, turning blood donation into a habit [8].

In China, a survey conducted in three cities, in Hubei Province, found that 75.5% of blood donors donated blood gratuitously to ‘help others’ whereas 12.9% did so in order to obtain free blood supply for themselves and their families in the future; 6.3% did so for social recognition and 2.8% for a free physical examination [9]. There is a dearth of qualitative studies on the topic in literature, especially those conducted in Mainland China [10-12]. Furthermore, there is an ongoing debate on whether blood should be regarded as a gift or as a commodity [13-15]. In general, the principle of voluntary blood donation campaigns in China is trying to avoid compulsory mobilization and the commercial blood supply, but promote voluntary, gratuitous, citizenship as the basis of blood donation [5].

Altruism is commonly cited as the primary motivation of blood donation but the picture may have been oversimplified. Titmuss contended that blood donors do not require or expect a return, and no one expects himself/herself (or his/her family) to accept free blood transfusion in the future [13]. Voluntary blood donation has hence been described as ‘perhaps the purest example’ of altruistic behaviors [16]. However, blood donation can also be beneficial to the donors and their family members. Symbolic incentives are also important [17]. Therefore, the reality requires a more comprehensive examination of incentives behind blood donation in China. The importance of the structural and institutionalized means to manage and to deliver such incentives has been overlooked. Social structures and institutions within a given society are supposed to inspire altruism. Merton suggests that institutionalized altruistic behavior leads to behaviors orientated to helping others through structured mechanisms to distribute rewards and penalties. The social structure would increase the frequency of individual altruistic behaviors, whether motives are altruistic or egotistic in nature [18]. It has been further argued that the social organization and the way of structuring social institutions, especially institutions concerning health and welfare, can encourage or discourage people’s motives for altruism [13].

China, as a country with a population of 1.37 billion [19] is in urgent need to develop an effective voluntary blood donation system to ensure a steady supply of blood for clinical use. There have been reports of shortage in the country and the number of donors need to be increased sharply [12,20]. The country is therefore actively formulating and updating its policy on blood donation. There is however, a dearth of data looking at the in-depth reasons and motivations behind blood donation. Such information is warranted to improve the current blood collection system. This study is therefore a timely one to examine the role of blood donation institutions in shaping public altruism.

One caution has to be made when promoting voluntary blood donation. In many countries or regions, previous studies have shown that some high risk individuals use blood donation as a means of testing stigmatized blood-borne infectious diseases, such as HIV [13,21-23]. Despite numerous measures such as counseling, advance blood test technology, and even penalties to safeguard contamination in blood donation, problems such as window period for detection still exist [24]. Therefore, health workers need to keep such considerations in mind when promoting blood donation.

## Methods

This study was conducted from May to October 2008, in Changsha, Hunan province, China. Changsha was chosen as its blood donation system typifies current models of metropolitan cities of China. In Changsha, the collection of whole blood is the dominate objective while apheresis platelets are collected to a significantly lesser extent. In June 2005 the amount of compensated blood supply decreased to zero; compulsory blood donation organized by employers and donation centers [1,20] was also abolished in 2007. Similar trends occurred in most major cities of China.

In this study, participatory observations were conducted at two blood donation stations and four blood collecting vehicles. In-depth interviews were conducted with 17 blood centers’ staff, and 58 blood donors at the aforementioned venues. After briefing about the objectives of the study, informed consent was obtained from all participants, who were assured of confidentiality, the use of pseudonyms, and safe storage of the data. All of the interviews and participant observations were conducted by the first author. Ethical approval was obtained from the department of anthropology at Sun Yat-sen University (NO.:FWA00007867).

The audio-recorded interview data were transcribed verbatim to capture cultural concepts and nuances embedded in the language, while another two members of the research team, with qualitative and ethnographic training, reviewed the transcripts to ensure accuracy. An independent audit trial was kept for any discrepancies from the verbal data to be compared with the notes of the audit trial. All transcriptions were coded by coders who first familiarized themselves with the raw information and developed a draft code list independently to check consistency with the data.

Continuously, new codes were added and unfit codes were discarded, keeping data grounded and developing content sensitivity. In the final process of the analysis, two investigators compared their code lists together to resolve any discrepancies. Final main codes were determined according to the common themes agreed [25]. All the above steps were undertaken by using software for qualitative data processing and analysis (ATLAS.ti 5.2). Key findings were then translated into English by an independent bilingual translator who was not a member of the research team. Upon conclusion of this process another independent bilingual back-translated the new English version into Chinese then compared his version with the original Chinese version and compared the previous.

## Results

The main themes found were policy-driven incentives, symbolic incentives, information feedback and role models, which constituted the system of institutional incentives.

### Insured blood use as a policy-driven institutionalized incentive

Blood recipients testified the need to pay for their blood transfusions, and reported the cost could be waived totally or partially for future blood needs for the donors and their family members. This incentive is an insurance in nature because not every blood donor, nor his/her family members, would necessarily draw on this opportunity over their lifetime. Testimony to contingent incentives is provided in the following.

L, a male Grade 3 university student, who donated blood for the first time on New Year’s Day of 2006, spoke about the donation policy, as well as his family’s attitudes.

When I donated blood then, I didn’t dare to tell my parents. For this reason, I learned more about the benefits of blood donation, because telling more benefits to my parents might make them feel better. I first told them that after the blood donation, anyone of the family could use blood for free just in case. They thought over and approved of my donation since this had no harm to my health…They donated blood later, too. As you know, few people would donate blood in my small county town.’ (20080521—Student L)

In this case, the blood reimbursement policy appeared to have served as an important incentive. In student L’s testimony, the incentive becomes a type of ‘insurance’ for blood users, especially for those from underprivileged families.

Another testimony was provided by Uncle B who lived in the countryside of XX City, Hunan Province. A doctor in Changsha told him that his daughter needed a blood transfusion. However, Uncle B was unable to afford this. The doctor told him to donate blood in order to use blood for free. As follows:

Q: ‘Uncle, how old are you?’

A: ‘57 years. I was born in 1951.’

Q: ‘You’re ineligible for blood donation because of your age. The prevailing blood donation age is from 18 to 55 years. Do you have any other relatives in your family?’

A: ‘My wife is as old as me. My daughter is 26 and eligible for donation, but she cannot do this. Oho. (After a while of silence) I had to persuade the doctor to let me donate blood.’

The uncle negotiated with the nurse but failed. He got off the blood collection vehicle and told me, ‘This policy is good, but why should there be an age limit?’ (20080625—Uncle B)

Secretary Y of Changsha Blood Center was interviewed at the blood center, claiming:

*‘In 2006, a patient who had donated 900 cc of blood (which means that he can use an unlimited amount of blood throughout his life) needed to be transfused with a large amount of blood. The cost was about RMB 20,*000 (US$3200). When *our director sent the money to him at his bed (as a reimbursement), his family was highly moved.’ (20080605—Secretary Y)*

Some donors interviewed however did not know about this policy, nor believed that they would need a blood transfusion. Mr. W worked in a factory which was near a blood donation center and said:

‘I did not know about this in the past, but a nurse just told me, just as what the poster says.’ ‘Hum, this policy is fairly good.’

Q: ‘Will you donate blood next time?’

A: ‘Of course, as long as I have time.’

Q: ‘Are you motivated by this blood use policy?’

After thinking for a while, he answered, *‘Maybe a little, but I do not come here for this. Look at me, how healthy I am! How can I use blood now?…’(20080628—Mr. W)*

Though some donors donated blood for reasons other than the insured blood incentive, they did not object to the policy. They stated, ‘It is better to have this policy in place’, thus being socially illustrative of the operationalization of an institutionalized blood donation mechanism.

### Symbolic meaning as an institutionalized incentive

Intangible symbolic incentives to donate blood included respect, care, meaning and the association with positive values. Such incentives fulfilled people’s social and mental health needs, as opposed to their materialistic or utilitarian needs, and in some cases may well inform their intention to donate blood. We contend here that such incentives are further institutionalized by such potent reinforcements as special souvenirs. In Changsha, blood donors received special souvenirs such as key holders, lovers’ watches, and T-shirts. The blood donation card was also reported by some informants to constitute a valuable souvenir. All these souvenirs bore the slogan ‘blood donation is beneficial to health and blood saves lives’, and the label of ‘Changsha Blood Center’. These souvenirs acted as symbolic interpretations of the potency of voluntary blood donation. In Changsha other examples of the gift provision were Olympic Fuwa dolls made specifically for the Beijing Olympic Game which represented the Olympic spirit. Some special editions were stenciled with an Olympic special memorial for gratuitous blood donation and were given to blood donors as a significant souvenir.

X, was Grade 3 university student, and had donated blood for the first time when she was a first year student and had subsequently donated blood four more times. We observed her attending to the donation center with a pile of blood donation cards to claim the Fuwa dolls. When she was asked about reasons for possessing so many blood donation cards, she said:

‘Sometimes when I saw a blood collecting vehicle on the street and I was due for blood donation, I then donated blood. Although I did not take any former blood donation card with me, I thought that it was good to have one more (smiling). I plan to get more cards before graduation.’ She told me that she would present this set of the Fuwa dolls to her boyfriend, because her boyfriend was ineligible for blood donation (highly myopic); he accompanied her during each blood donation. She said, ‘This is our shared gift.’ When I asked her if she valued these small gifts, she said: ‘It is of course good to have them, just for memory. In fact, every donor does not care about any reward, and a small souvenir would be okay, such as a blood donation card.’ (20080612—Student X)

At noon time on June 10, three male and two female students arrived at the donation vehicle in their school uniforms and were each given a blood donation card. One of the boys then said:

‘We don’t need so many blood donation cards. Can you just give us one card that bears all our names?’ I told him that one blood donation card was for one person only and his request was impossible. He added, ‘Why don’t you put our barcodes (used for blood donor identification) on one card?’ Feeling surprised, I asked him why. A girl answered, ‘We just need a memorial of our friendship, because we come here together.’ ‘Can’t one card per person also be taken as a memorial?’ I gave them this advice. However, that boy said, ‘This cannot represent our friendship.’ Seeing that they insisted, I did not want to frustrate them and told their request to the nurse. Finally, these students did not succeed. (20080610—several senior high school students).

Such a testimony suggests that in China, blood is highly symbolic and carries special cultural associations, such as a bond of loyalty and friendship. In China in historic times, people were sworn into ceremonies for joining specific associations by shedding blood into a bowl of wine and sharing it to symbolize fraternity [26]. Inherent in these illustrations are the Chinese notions of ‘gloriousness’ and symbolic meanings of owning a blood donation card. Merton suggests that, this (institutionalized incentive) helps increase the number of individuals choosing altruistic actions and goes far beyond any other possible action (such as actions based completely on human nature, early-stage socialization or any other disposition) [18]. In our ethnography, we observed that monetary and utilitarian compensation had been replaced by such institutionalized symbolic incentives, which was a significant transformation.

### Information about health status and positive self-appraisal as institutionalized incentives to donate

According to our observation, information about the donor’s health status has been used as an institutionalized incentive to promote voluntary blood donation. To ensure blood safety, the donated blood specimens are tested on-site for HBsAg using a rapid screening test. Laboratory testing for HIV antibodies, HCV antibodies, HBsAg, syphilis, HB, ALT, and blood types are also performed; those blood specimens with positive testing results will be discarded. Under those circumstances, the donors will be informed about the positive results and in the case of a positive HIV testing result, the case will also be reported to the local CDC for follow-up. Some CDCs go further to inform the donors that their testing results are all negative. It is then possible for donors to use blood donation as a means of testing stigmatized blood-borne infectious diseases such as HIV and syphilis. In the study conducted we mentioned, the percentage is relatively low (2.8% of donors are for a free physical examination) [9], but caution is required.

In May 2007, the Changsha Blood Center established a Short Message Service (SMS) system to promote voluntary blood donation. Director J of the Public Services Section explained that blood donors were very concerned about the personal implication of the afore-mentioned testing results. He explained:

‘This is a feedback. The blood donation cannot just end like this. It is stated on the back of the blood donation card that we can call the toll-free hotline to inquire about blood information. Mobile phones are very popular today, and we set up this platform just to communicate physical examination information to blood donors and enable them to know their physical conditions’ (20080527—Director J).

Therefore, testing results have been used as an institutionalized incentive, however the practice is debatable as it may increase the number of high risk people using blood donation as a means of testing. The potential increase in the number of donors needs to be weighed against the risk of contamination in the blood supply due to the window period for detection of blood-borne infectious diseases such as HIV/AIDS.

The current system installs multiple measures to safeguard blood safety besides the laboratory tests aforementioned. First, donors need to fill out a form to declare that they have not been diagnosed with a list of blood-borne diseases such as leprosy and anaphylactic disease, and that they do not belong to high risk groups (e.g. men who have sex with men, drug users, those with multiple sex partners and suspected HIV/STD status). Second, their name and identification will be checked against a database of individuals previously found to be unsuitable for blood donation. Third, a nurse will provide counseling to remind them about the window period property and that they should not donate blood if they belong to the higher risk categories. Forth, they can withdraw the blood donated if they believe some risks exist and a contact number will be given to them. Fifth, they are told that positive HIV results will be reported to the local CDC and they will be contacted by the CDC if their testing results are found to be HIV positive. Last, they are reminded that there are centers providing free HIV and syphilis testing and there may be penalties if they provide false information in the form that they fill out. Although the preventive procedures are quite comprehensive, we believe that this particular incentive of informing donors about negative testing results should not be actively promoted. Furthermore, health education for prospective donors is of utmost importance.

In addition to the testing-related incentive, the SMS however, also served as an institutionalized channel for communication between the blood center and donors. This in turn, reinforced the act of donation by instilling a sense of identification and social belongingness, hence furthering positive self-appraisal. It should therefore be maintained. Testimony is as follows:

‘However, this platform has more functions now. On festivals and birthdays, we would send short messages to blood donors to show our support and send our blessings. With such respect, blood donors will surely feel comfortable. Only in this way they will be motivated to donate blood. In addition, when our blood bank lacks blood, we would send messages to them to let them know what types of blood we are short of. They would come here whenever they are free. For example, before this Spring Festival, we were short of blood and sent short messages. Within 4 days, there were over 700 donors.’ (20080527—Director J)

Some blood donors further reiterated positive associations from the act of blood donation [27]:

‘I think it is a pleasant and meaningful thing to rescue patients who need blood urgently with my weak force.’—Ms. H

‘Beautiful angels, thank you for your hard work! Everyone should do its best to help those who need help.’—Ms. Z

‘This is my honor because it is my purpose to help others, just like doctors who heal the wounded and rescue the dying. Thank you for giving me this opportunity. I also believe that the wounded person will recover.’—Ms. S

‘Thank Changsha Blood Center very much for your birthday blessings to me. Although I used to celebrate birthdays on the lunar calendar other than the Gregorian calendar, I still feel the same happiness! Blood donation is something compulsory for me, and I will keep doing this in the rest of my life, because this is very meaningful. Thank you again for your blessings’—Mr. C

We can see from these messages that blood donors interviewed in this study in Changsha had framed voluntary blood donation positively as an altruistic and honorable behavior. Participation in such ‘good’ behaviors was seen to bring about positive self-appraisal, which together with the information about one’s disease status, may well serve as incentives institutionalized by the SMS. The use of SMS’s further enabled interactions between the blood center and blood donors are supported.

### Identification with role models as an institutionalized incentive

In China both at national and city levels, celebrities and idols are used as blood donation ambassadors. These honorary role models serve to remind the general public that donating blood is a desirable way of contributing to public altruism.

‘In 2007, we invited famous host WH of our provincial TV station’s economic life channel as our province’s image ambassador for gratuitous blood donation. Now, he is featured in all of our brochures and posters. In our city (Changsha), two ambassadors have been invited. One is a host of the economic radio channel, called ZQH, who is favored by young people and also an Olympic torchbearer. Another one was TX, one of the Top 10 Super Girls. These celebrities are very popular among the youth, and are very helpful to our publicity. They will attend the June 14th event (World Blood Donor Day) in person.’ (20080605—Secretary Y)

Currently in China state leaders have become ‘shadow ambassadors’ of the blood donation campaign. For instance, photos of President Hu Jintao and the Minister of Health, Chen Zhu, donating blood were placed in some blood donation centers’ websites, with captions like ‘Leading by Example’. By donating blood in person, state leaders and local officials are communicating the message that voluntary blood donation is a ‘great and glorious’ behavior which brings public good ,and is highly advocated, encouraged and endorsed by the state. In China the identification with these role models becomes an incentive as it reinforces one’s self-image of being a solid good citizen, and creates a positive feeling of being drawn closer to the idols by following their steps. These interpretations would be obvious in the following testimony.

Director W who was responsible for donor recruitment at the blood center said:

‘City leaders, the municipal government and the municipal civilization office support our work greatly. The propaganda department of the municipal Party committee came here last year (2007). When our volunteer service team was founded, the head of the propaganda department of the municipal Party committee was present in person. These are a great encouragement for us.’ (20080605—Director W)

When summing up the blood center’s publicity experience, Director J of the Public Services Section said emotionally:

‘I still think that the concern of the leadership is the most important. With their concern, the gratuitous blood donation program is at least half successful. If the city’s leadership regards this as a political achievement, this will be half successful. In fact, their concern is an intangible form of leadership, because the image of the government is irreplaceable.’ (20080527—Director J)

In the above testimony identification with such role models through blood donation became another institutionalized incentive for blood donation.

## Discussion and conclusions

This paper has examined Chinese public blood donation behaviors from an institutional perspective. The focus has been the interactions between the public and blood collection organizations/institutions. We should continue with the current nationwide campaigns that publicize and emphasize the voluntary aspect of blood donation because such blood collection pattern has been proven feasible in both China and other countries. The strong community spirit is a good basis for developing a robust system of volunteerism in China. Secondly, the public utility of drawing on the concept of altruism should be integrated with the diversified motives for blood donations found in the reported study. Furthermore, of importance is that the Chinese public has become sensitized to blood donation as not only helpful to others but also good for oneself and society in the long run. Thus the institutionalization of instructional incentives should become an explicit mechanism in the current blood donation in China. Collectivity and the strong administrative system in mainland of China are very helpful to create these incentivized systems.

Further inter-disciplinary research is needed to explore the values attached to different types of incentives and to develop new ones, as well as ways to institutionalize these new incentives. Examples of potential institutionalized incentives include symbols of fraternity associated with group donation in schools and social organizations and improvement in corporate image of social responsibility. Furthermore, ritual of passage for adulthood or college graduation and memories for special anniversaries require sustained advocacy. Another way to reinforce the public altruistic impulse is to bring blood donors information about the recovering recipients of local transfusions through newsletters or film loops in blood collection point waiting rooms and vans. Such feedback helps people connect with the real human meaning of their gifted impulse. The exemplary effect of celebrities should be utilized to emphasize the common citizenship aspects of blood donation, and further sustain and enhance the current voluntary blood donation initiatives. New role models may also be recruited from different communities such as that of teachers, health professionals, and corporate businesses in order to generate incentives that are more specific to the community. Of paramount importance blood collection organizations need to clarify their current procedures for public communicating strategies and policies in order for the public to better understand the blood donation program. Like any other products, the institutionalized incentives, new and old, need to be marketed to prospective donors.

Finally, caution is required to educate donors that those with risk behaviors related to HIV or other blood-borne infectious diseases should not donate their blood due to the risk of blood contamination during the window period for detection [3,4]. Currently, preventive measures involve counseling and laboratory screening and other procedures. Donors have also been advised that they can take up free HIV testing provided by the CDC. However, such an institutionalized incentive may increase the risk to use blood donation as a means of testing for stigmatized blood-borne infectious diseases. For instance, some high risk people may see it as a less stigmatized form of HIV testing. This challenge is almost universal as the screening for some blood borne diseases are necessary and cannot be removed from the blood donation process while prospective donors know about such screening tests. The situation does not seem to be very serious but it could get worse if the testing aspect of blood donation is actively promoted. Although we are unable to abolish such screening tests, health educators should hence not promote such testing actively as an incentive for blood donation. Other useful measures include referrals and close collaboration between blood donation centers and user-friendly non-governmental organizations experienced in providing free HIV testing to high risk individuals.

Cautions should also be made when promoting testing in group settings, as some high-risk individuals may donate blood due to conformity. It should be emphasized that donations are totally voluntary. Those who are temporarily unfit (e.g. having a cold, lack of sleep or mild illnesses) will not be accepted to donate blood; such reasons should be publicized so that they can be used as justification if needed to minimize the effect of group conformity. It should be publicized that here is a hot-line for immediate confidential withdrawal after blood donation has been made in such settings. Such measures should minimize the risk of contamination via those settings. Overall, it is important to educate potential donors about the concepts of window period and blood contamination, to provide alternatives and to instill a strong sense of social responsibility to protect others. Ultimately, the integrity of the donors is important. According to Titmuss, only in the voluntary and unpaid blood donation system, the honesty to report one’s health condition could be guaranteed, which would most likely increase the quality of blood and reduce the risk of contamination [13].

We acknowledge that the results of this study may not be generalized to the Chinese blood donation system as a whole, given it was conducted only in one city and it was comprised of such contextual data as opposed to larger epidemiological work. Despite such limitations, this study provides rich culturally informed data on institutional blood donation incentives that contribute to our understanding of the relationship between institutional incentives and the voluntary blood donation of contemporary Chinese citizens. This study also informs an important knowledge gap of the new public health campaigns that advocate for blood donation behaviors in China. Furthermore, insights have hence been highlighted that add support for evaluated social marketing exercises for specific types of institutionalized incentives to generate evidence-based public health strategies for promoting blood donation in China.

## Competing interests

The authors declare that they have no competing interests.

## Authors’ contributions

CY wrote the first draft and the final version. YC assisted with the collection of literature. EH reviewed the draft of the paper and made major revisions of the paper. JTF Lau reviewed the paper and finalized it with CY. All authors read and approved the final manuscript.

## Pre-publication history

The pre-publication history for this paper can be accessed here:

http://www.biomedcentral.com/1471-2458/13/524/prepub
